# Supplementary material for: Natural Populations from the Phytophthora palustris Complex Show a High Diversity and Abundance of ssRNA and dsRNA Viruses
Source: J Fungi (Basel). 2022 Oct 24;8(11):1118. doi: 10.3390/jof8111118 (PMC9698713; doi:10.3390/jof8111118)
Supplement: Supplementary file 1 [file jof-08-01118-s001.zip › Supplementary figures.pdf]

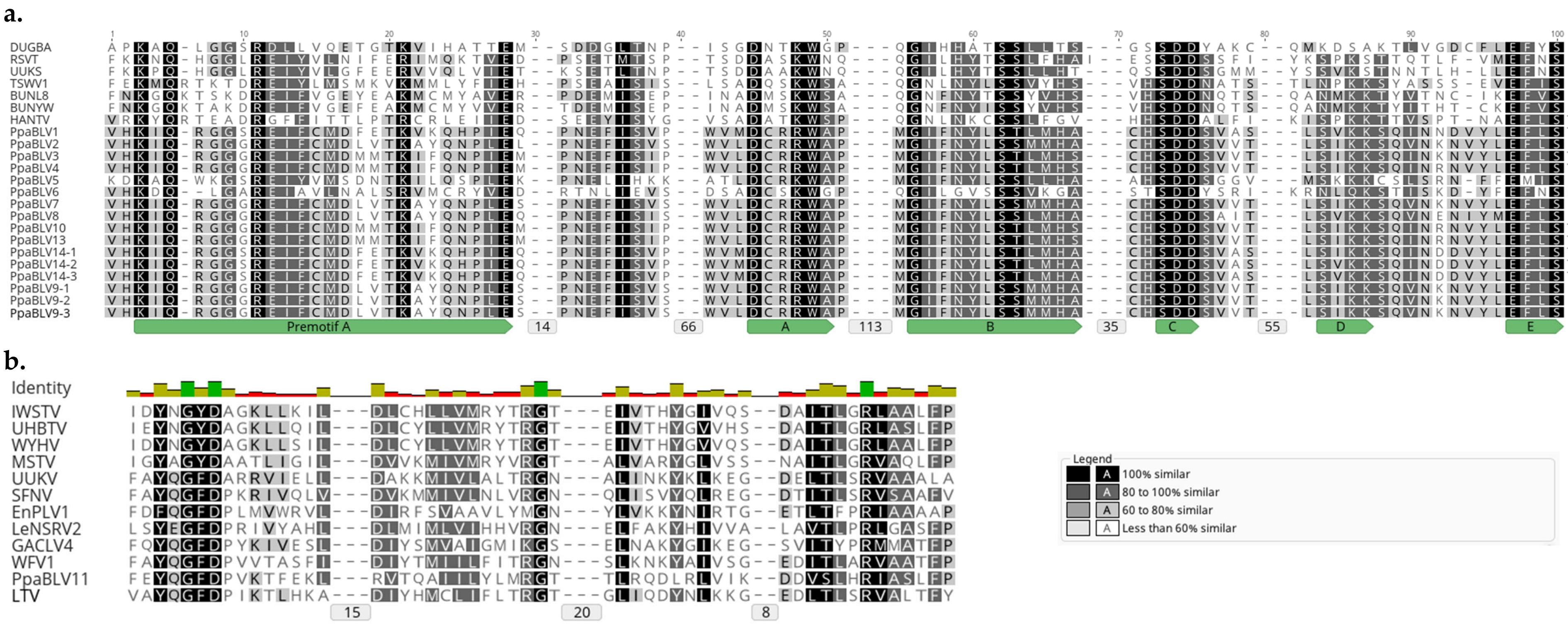

Figure S1. a. Amino acid alignment showing Bunya_RdRp conserved motifs A to E and premotif A within the RdRp of PpaBLV1-14 and selected bunyaviruses. DUGBA, Dugbe virus (accession number Q66431); RSVT, Rice stripe virus (Q85431); UUKS, Uukuniemi virus S23 (P33453); TSWV, Tomato spotted wilt virus (P28976); BUNYW, Bunyawera virus (P20470); BUNL8, La Crosse virus L78 (Q8JPR2); HANTV, Hantaan virus 76-118 (P23456). Gray boxes with numbers represent the number of positions deleted in the MUSCLE alignment. **b**. Amino acid alignment showing the conserved motifs of *Tenuivirus*/*Phlebovirus* nucleocapsid protein within PpaBLV11 and selected bunyaviruses. IWSTV, Iranian wheat stripe tenuivirus (Q7TBL7); UHBTV, Urochloa hoja blanca tenuivirus (O11440); WYHV, Wheat yellow head virus (P84254); MSTV, Maize stripe tenuivirus (Q50HQ8); UUKV, Uukuniemi virus S23 (P22025); SFNV, Sandfly fever Naples virus (NC_006318); EnPLV1, Entoleuca phenui-like virus 1 (NC_055436); LeNSRV2, Lentinula edodes negative-strand RNA virus 2 (YP_010086263); GACLV4, Grapevine associated cogu-like virus 4 (QMP81965); LTV, Lihan tick virus (QFR36186). WFV1, Wuhan Fly Virus 1 (YP_009304999). Gray boxes with numbers represent the number of positions deleted in the MUSCLE alignment. The % of similarities are calculated based on Blosum62 score matrix with a threshold of 1.

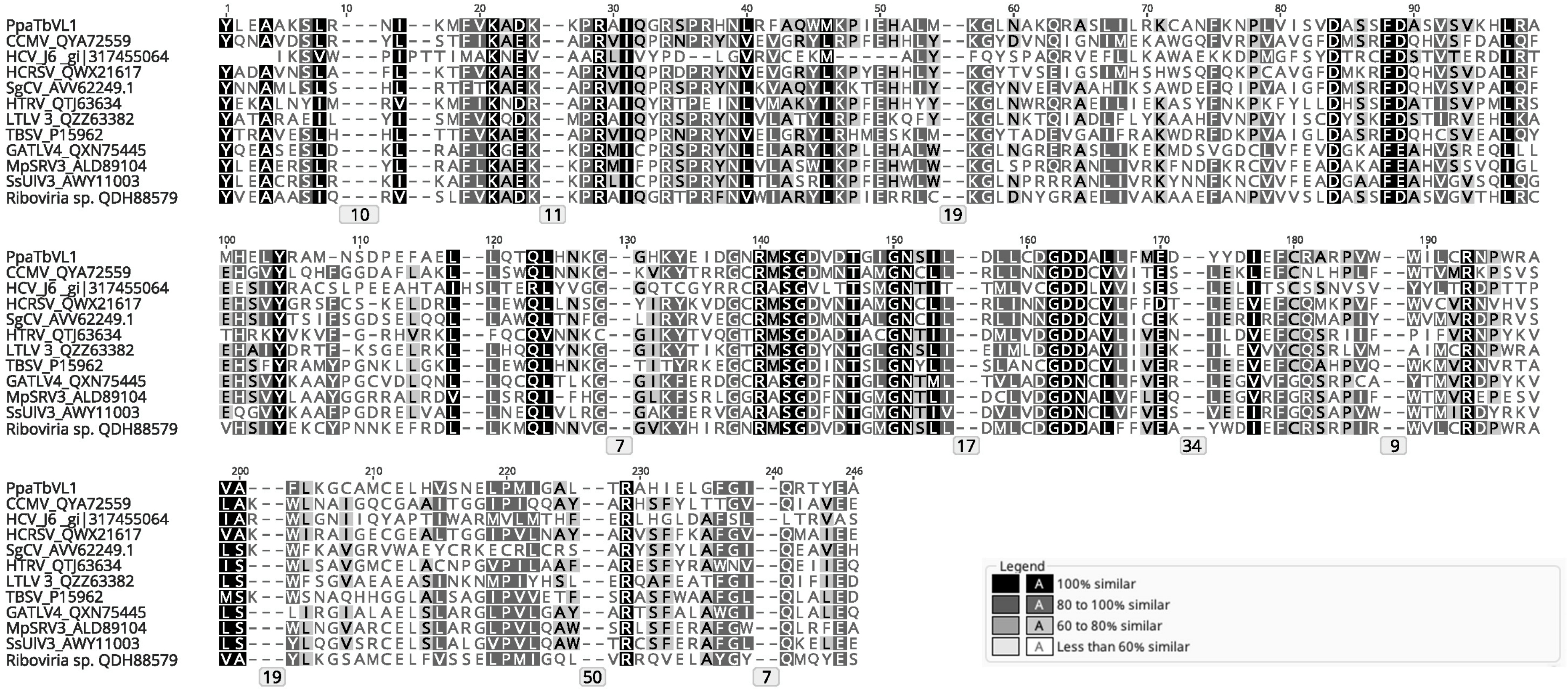

Figure S2. Amino acid alignment showing the conserved RdRp conserved motifs of PpaTbVL1 and selected tombusviruses. CCMV, Cowpea mottle virus (QYA72559); HCV-J6, Hepacivirus C (4ADP_A); HCRSV, Hibiscus chlorotic ringspot virus (QWX21617); SgCV, Saguaro cactus virus (AVV62249); HTRV, Hymenopteran tombus-related virus (QTJ63634); LTLV3, Leuven Tombus-like virus 3 (QZZ63382); TBSV, Tomato bushy stunt virus (P15962); GATLV4, Grapevine-associated tombus-like virus 4 (QXN75445); MpSRV3, Macrophomina phaseolina single-stranded RNA virus 3 (ALD89104); SsUIV3, Sclerotinia sclerotiorum umbra-like virus 3 (AWY11003); Riboviria sp., RNA-dependent RNA polymerase (QDH88579). Gray boxes with numbers represent the number of positions deleted in the MUSCLE alignment. The % of similarities are calculated based on Blosum62 score matrix with a threshold of 1.

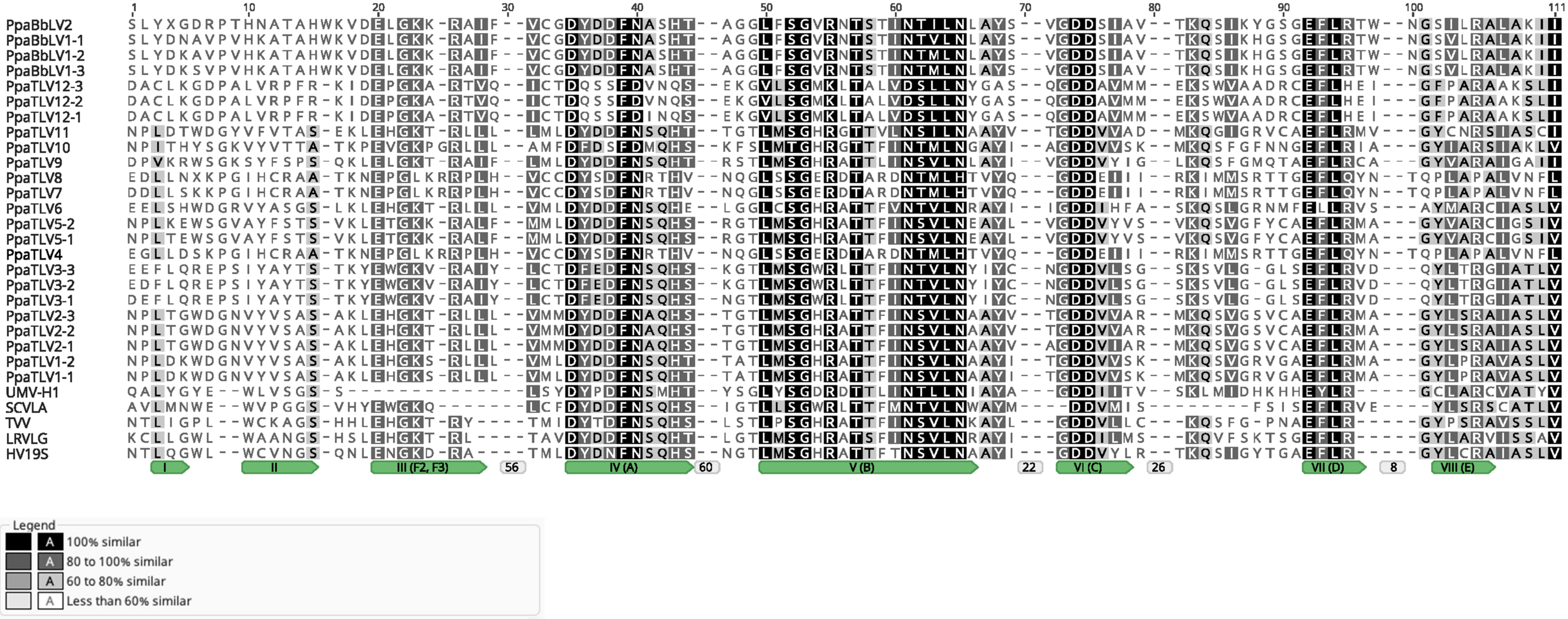

Figure S3. Conserved aa sequence motifs (I-VIII) of RdRp of totiviruses and botybirnaviruses in *P. palustris*. **PpaBbLV1-2** and **PpaTLV1-**12; **UMVH1**, Ustilago maydis virus H1 (NP_620728); **SCVLA**, Sacharomyces cerevisiae virus L-A (Q87025); **TVV**, Trichomonas vaginalis virus 3 (Q8V615); **LRVLG**, Leishmania RNA virus 1 (Q02382); **HV19S**, Helminthosporium victoriae virus 190S (O57044). Gray boxes with numbers represent the number of positions deleted in the MUSCLE alignment. The % of similarities are calculated based on Blosum62 score matrix with a threshold of 1.

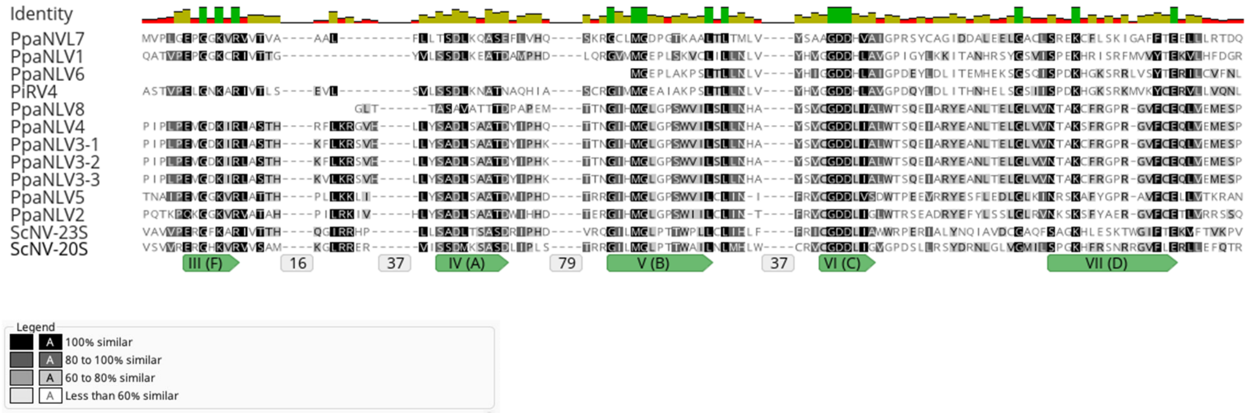

Figure S4. Conserved aa sequence motifs (III-VII) of RdRp of narna-like viruses in *P. palustris* and **ScNV-23S**, Saccharomyces 23S RNA narnavirus (NP660177), **ScNV-20S,** Saccharomyces 20S RNA narnavirus (NP660178); **PiRV4**, Phytophthora infestans RNA virus 4 (YP009241365). Gray boxes with numbers represent the number of positions deleted in the MUSCLE alignment. The % of similarities are calculated based on Blosum62 score matrix with a threshold of 1.

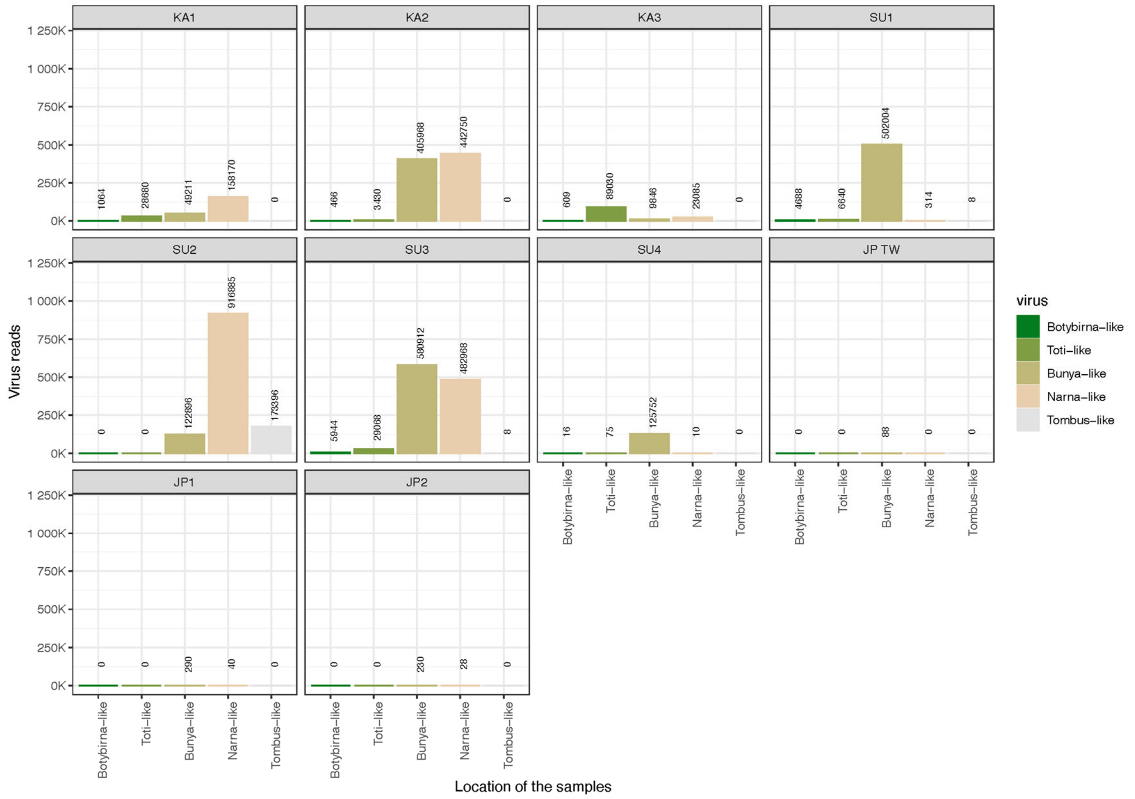

Figure S5. Contingency graphs showing the number of viral reads per virus group obtained in each RNA library.
