# Supplementary material for: Natural Populations from the Phytophthora palustris Complex Show a High Diversity and Abundance of ssRNA and dsRNA Viruses
Source: J Fungi (Basel). 2022 Oct 24;8(11):1118. doi: 10.3390/jof8111118 (PMC9698713; doi:10.3390/jof8111118)
Supplement: Supplementary file 1 [file jof-08-01118-s001.zip › Supplementary tables.pdf]

Supplementary tables

Table S1. List of '*Phytophthora palustris*' isolates used in the present study.

Isolate Code	Island	Site	Latitude	Longitude	Ecosystem	Host	Collecting year	RNA POOL
**JP1654**	**Japan**	JP_F32	28.495226	129.661421	Forest stream	Fallen leaves	2018	**JP 2**
**JP1201**	**Japan**	JP_R31	31.82887	130.64113	Forest stream	Fallen leaves	2018	**JP 1**
**JP1268**	**Japan**	JP_R35	28.39768	129.59153	Forest stream	Fallen leaves	2018	**JP 1**
**JP1257**	**Japan**	JP_R35	28.39768	129.59153	Forest stream	Fallen leaves	2018	**TW-JP**
**JP1287**	**Japan**	JP_R36	28.399477	129.591249	Forest stream	Fallen leaves	2018	**JP 1**
**JP1271**	**Japan**	JP_R36	28.399477	129.591249	Forest stream	Fallen leaves	2018	**TW-JP**
**JP1301**	**Japan**	JP_R37	28.3933	129.594529	Forest stream	Fallen leaves	2018	**JP 1**
**JP1319**	**Japan**	JP_R39	28.434254	129.589337	Forest stream	Fallen leaves	2018	**JP 1**
**JP1321**	**Japan**	JP_R40	28.495226	129.661421	Forest stream	Fallen leaves	2018	**JP 1**
**JP1324**	**Japan**	JP_R40	28.495226	129.661421	Forest stream	Fallen leaves	2018	**JP 1**
**JP1338**	**Japan**	JP_R40	28.495226	129.661421	Forest stream	Fallen leaves	2018	**TW-JP**
**JP1350**	**Japan**	JP_R41	28.477746	129.675774	Forest stream	Fallen leaves	2018	**JP 1**
**JP1355**	**Japan**	JP_R42	28.329501	129.481019	Forest stream	Fallen leaves	2018	**TW-JP**
**JP1362**	**Japan**	JP_R43	28.289778	129.434379	Forest stream	Fallen leaves	2018	**JP 1**
**JP1370**	**Japan**	JP_R44	28.22532	129.368641	Forest stream	Fallen leaves	2018	**JP 2**
**JP1379**	**Japan**	JP_R44	28.22532	129.368641	Forest stream	Fallen leaves	2018	**JP 2**

Isolate Code	Island	Site	Latitude	Longitude	Ecosystem	Host	Collecting year	RNA POOL
**JP1389**	**Japan**	JP_R46	28.377835	129.458181	Forest stream	Fallen leaves	2018	**JP 2**
**JP1509**	**Japan**	JP_R47	26.58825	128.006573	Forest stream	Fallen leaves	2018	**JP 2**
**JP1511**	**Japan**	JP_R47	26.58825	128.006573	Forest stream	Fallen leaves	2018	**JP 2**
**JP1523**	**Japan**	JP_R48	26.759784	128.218436	Forest stream	Fallen leaves	2018	**TW-JP**
**JP1533**	**Japan**	JP_R49	26.759506	128.216925	Forest stream	Fallen leaves	2018	**JP 2**
**JP1534**	**Japan**	JP_R49	26.759506	128.216925	Forest stream	Fallen leaves	2018	**JP 2**
**JP1566**	**Japan**	JP_R50	26.509577	127.904906	Forest stream	Fallen leaves	2018	**JP 2**
**JP1543**	**Japan**	JP_R50	26.509577	127.904906	Forest stream	Fallen leaves	2018	**TW-JP**
**JP1564**	**Japan**	JP_R50	26.509577	127.904906	Forest stream	Fallen leaves	2018	**TW-JP**
**KA0033**	**Kalimantan**	KA_F13	0.88671	116.66049	Forest stream	Fallen leaves	2019	**KA 3**
**KA0044**	**Kalimantan**	KA_R03	4.06809	117.16497	River	Fallen leaves	2019	**KA 1**
**KA0085**	**Kalimantan**	KA_R03	4.06809	117.16497	River	Fallen leaves	2019	**KA 1**
**KA0093**	**Kalimantan**	KA_R03	4.06809	117.16497	River	Fallen leaves	2019	**KA 1**
**KA0046**	**Kalimantan**	KA_R03	4.06809	117.16497	River	Fallen leaves	2019	**KA 2**
**KA0052**	**Kalimantan**	KA_R03	4.06809	117.16497	River	Fallen leaves	2019	**KA 2**
**KA0059**	**Kalimantan**	KA_R03	4.06809	117.16497	River	Fallen leaves	2019	**KA 2**
**KA0081**	**Kalimantan**	KA_R03	4.06809	117.16497	River	Fallen leaves	2019	**KA 2**
**KA0092**	**Kalimantan**	KA_R03	4.06809	117.16497	River	Fallen leaves	2019	**KA 2**
**KA0106**	**Kalimantan**	KA_R05	4.04382	117.043776	River	Fallen leaves	2019	**KA 1**
**KA0109**	**Kalimantan**	KA_R05	4.04382	117.043776	River	Fallen leaves	2019	**KA 1**
**KA0110**	**Kalimantan**	KA_R05	4.04382	117.043776	River	Fallen leaves	2019	**KA 1**
**KA0127**	**Kalimantan**	KA_R05	4.04382	117.043776	River	Fallen leaves	2019	**KA 1**
**KA0129**	**Kalimantan**	KA_R05	4.04382	117.043776	River	Fallen leaves	2019	**KA 1**
**KA0101**	**Kalimantan**	KA_R05	4.04382	117.043776	River	Fallen leaves	2019	**KA 2**
**KA0102**	**Kalimantan**	KA_R05	4.04382	117.043776	River	Fallen leaves	2019	**KA 2**
**KA0116**	**Kalimantan**	KA_R05	4.04382	117.043776	River	Fallen leaves	2019	**KA 2**
**KA0119**	**Kalimantan**	KA_R05	4.04382	117.043776	River	Fallen leaves	2019	**KA 2**
**KA0120**	**Kalimantan**	KA_R05	4.04382	117.043776	River	Fallen leaves	2019	**KA 2**
**KA0134**	**Kalimantan**	KA_R06	3.82856	116.79734	River	Fallen leaves	2019	**KA 1**
**KA0139**	**Kalimantan**	KA_R06	3.82856	116.79734	River	Fallen leaves	2019	**KA 1**

Isolate Code	Island	Site	Latitude	Longitude	Ecosystem	Host	Collecting year	RNA POOL
**KA0142**	**Kalimantan**	KA_R06	3.82856	116.79734	River	Fallen leaves	2019	**KA 1**
**KA0146**	**Kalimantan**	KA_R06	3.82856	116.79734	River	Fallen leaves	2019	**KA 3**
**KA0147**	**Kalimantan**	KA_R06	3.82856	116.79734	River	Fallen leaves	2019	**KA 3**
**KA0152**	**Kalimantan**	KA_R06	3.82856	116.79734	River	Fallen leaves	2019	**KA 3**
**KA0156**	**Kalimantan**	KA_R06	3.82856	116.79734	River	Fallen leaves	2019	**KA 3**
**KA0167**	**Kalimantan**	KA_R06	3.82856	116.79734	River	Fallen leaves	2019	**KA 3**
**KA0170**	**Kalimantan**	KA_R06	3.82856	116.79734	River	Fallen leaves	2019	**KA 3**
**KA0187**	**Kalimantan**	KA_R08	4.06809	117.16497	River	Fallen leaves	2019	**KA 3**
**KA0189**	**Kalimantan**	KA_R09	3.37549	117.04230	River	Fallen leaves	2019	**KA 1**
**SU0835**	**Sumatra**	SU_F11	0.08716	101.43640	Forest soil	Fallen leaves	2018	**SU 4**
**SU0839**	**Sumatra**	SU_F11	0.08716	101.43640	Forest soil	Fallen leaves	2018	**SU 4**
**SU0875**	**Sumatra**	SU_F17	-0.35439	101.80882	Forest soil	Baiting	2018	**SU 1**
**SU0982**	**Sumatra**	SU_F24	2.233063	99.204049	Forest soil	Baiting	2018	**SU 1**
**SU0987**	**Sumatra**	SU_F25	2.233063	99.204049	Forest soil	Baiting	2018	**SU 1**
**SU0777**	**Sumatra**	SU_F40	-0.59987	100.10982	Mangrove	Fallen leaves	2018	**SU 1**
**SU1361**	**Sumatra**	SU_F42	0.8711	102.34687	Mangrove	Fallen leaves in sea	2018	**SU 4**
**SU1370**	**Sumatra**	SU_F42	0.8711	102.34687	Mangrove	Fallen leaves in sea	2018	**SU 4**
**SU1477**	**Sumatra**	SU_N41	0.421098	101.865788	Pond in nursery	Fallen leaves	2018	**SU 1**
**SU1474**	**Sumatra**	SU_N41	0.421098	101.865788	Pond in nursery	Fallen leaves	2018	**SU 1**
**SU1452**	**Sumatra**	SU_N41	0.421098	101.865788	Nursery ditch	Fallen leaves	2018	**SU 4**
**SU0235**	**Sumatra**	SU_R01	0.03625	101.40635	River	Fallen leaves	2018	**SU 1**
**SU0245bTRUE**	**Sumatra**	SU_R02	0.04794	101.41029	River	Fallen leaves	2018	**SU 1**
**SU0249**	**Sumatra**	SU_R02	0.04794	101.41029	River	Fallen leaves	2018	**SU 1**
**SU0246**	**Sumatra**	SU_R02	0.04794	101.41029	River	Fallen leaves	2018	**SU 4**
**SU0015**	**Sumatra**	SU_R03	0.02641	101.45221	River	Fallen leaves	2018	**SU 1**
**SU0250**	**Sumatra**	SU_R03	0.02641	101.45221	River	Fallen leaves	2018	**SU 1**
**SU0264**	**Sumatra**	SU_R03	0.02641	101.45221	River	Fallen leaves	2018	**SU 1**
**SU0019**	**Sumatra**	SU_R04	0.02072	101.44307	River	Fallen leaves	2018	**SU 2**
**SU0274**	**Sumatra**	SU_R05	0.01545	101.50026	River	Fallen leaves	2018	**SU 4**
**SU0281**	**Sumatra**	SU_R05	0.01545	101.50026	River	Fallen leaves	2018	**SU 4**

Isolate Code	Island	Site	Latitude	Longitude	Ecosystem	Host	Collecting year	RNA POOL
**SU0043**	**Sumatra**	SU_R06	0.02612	101.49284	River	Fallen leaves	2018	**SU 2**
**SU0302**	**Sumatra**	SU_R06	0.02612	101.49284	River	Fallen leaves	2018	**SU 2**
**SU0046**	**Sumatra**	SU_R07	0.10733	101.46314	River	Fallen leaves	2018	**SU 2**
**SU0315**	**Sumatra**	SU_R07	0.10733	101.46314	River	Fallen leaves	2018	**SU 2**
**SU0317**	**Sumatra**	SU_R07	0.10733	101.46314	River	Fallen leaves	2018	**SU 2**
**SU0064**	**Sumatra**	SU_R08	0.10847	101.48398	River	Fallen leaves	2018	**SU 2**
**SU0072**	**Sumatra**	SU_R08	0.10847	101.48398	River	Fallen leaves	2018	**SU 2**
**SU0318**	**Sumatra**	SU_R08	0.10847	101.48398	River	Fallen leaves	2018	**SU 2**
**SU0394**	**Sumatra**	SU_R08	0.10847	101.48398	River	Fallen leaves	2018	**SU 2**
**SU0063**	**Sumatra**	SU_R09	0.09567	101.39207	River	Fallen leaves	2018	**SU 2**
**SU0326**	**Sumatra**	SU_R09	0.09567	101.39207	River	Fallen leaves	2018	**SU 2**
**SU0399**	**Sumatra**	SU_R10	0.07649	101.39411	River	Fallen leaves	2018	**SU 4**
**SU0344**	**Sumatra**	SU_R11	0.08716	101.43640	River	Fallen leaves	2018	**SU 3**
**SU0401**	**Sumatra**	SU_R11	0.08716	101.43640	River	Fallen leaves	2018	**SU 3**
**SU0095**	**Sumatra**	SU_R12	0.09272	101.42295	River	Fallen leaves	2018	**SU 3**
**SU0098**	**Sumatra**	SU_R12	0.09272	101.42295	River	Fallen leaves	2018	**SU 4**
**SU0353**	**Sumatra**	SU_R13	-0.35095	101.81692	River	Fallen leaves	2018	**SU 4**
**SU0135**	**Sumatra**	SU_R14	-0.34706	101.82067	River	Fallen leaves	2018	**SU 3**
**SU0141**	**Sumatra**	SU_R14	-0.34706	101.82067	River	Fallen leaves	2018	**SU 4**
**SU0409**	**Sumatra**	SU_R14	-0.34706	101.82067	River	Fallen leaves	2018	**SU 4**
**SU0158**	**Sumatra**	SU_R15	-0.37647	101.86426	River	Fallen leaves	2018	**SU 3**
**SU0160**	**Sumatra**	SU_R15	-0.37647	101.86426	River	Fallen leaves	2018	**SU 3**
**SU0361bTRUE**	**Sumatra**	SU_R15	-0.37647	101.86426	River	Fallen leaves	2018	**SU 3**
**SU0411**	**Sumatra**	SU_R16	-0.35697	101.84584	River	Fallen leaves	2018	**SU 3**
**SU0376**	**Sumatra**	SU_R17	-0.35439	101.80882	River	Fallen leaves	2018	**SU 3**
**SU0531**	**Sumatra**	SU_R24	2.23306	99.20444	River	Fallen leaves	2018	**SU 3**
**SU0575**	**Sumatra**	SU_R26	2.279201	99.189692	River	Fallen leaves	2018	**SU 3**
**SU0612**	**Sumatra**	SU_R29	2.711036	99.002634	River	Fallen leaves	2018	**SU 4**
**SU0618**	**Sumatra**	SU_R30	2.73353	99.045591	River	Fallen leaves	2018	**SU 3**
**SU0656**	**Sumatra**	SU_R34	0.94306	100.52000	River	Fallen leaves	2018	**SU 3**

Isolate Code	Island	Site	Latitude	Longitude	Ecosystem	Host	Collecting year	RNA POOL
**SU0696**	**Sumatra**	SU_R38	0.09139	100.69667	River	Fallen leaves	2018	**SU 3**
**TW436**	**Taiwan**	TW_F22	2.215452	120.83867	Forest Swamp soil	*Glochidion rubrum*	2013	**TW-JP**
**TW437**	**Taiwan**	TW_F22	2.215452	120.83868	Forest Swamp soil	*Glochidion rubrum*	2013	**TW-JP**
**TW438**	**Taiwan**	TW_F22	2.215452	120.83869	Forest Swamp soil	*Glochidion rubrum*	2013	**TW-JP**
**TW439**	**Taiwan**	TW_F22	2.215452	120.83870	Forest Swamp soil	*Glochidion rubrum*	2013	**TW-JP**
**TW440**	**Taiwan**	TW_F22	2.215452	116.79734	Forest Swamp soil	*Glochidion rubrum*	2013	**TW-JP**

Collectors of the isolates:
Taiwan - T. Jung, M. Horta Jung, C.-H. Fu, K.-L. Hsueh and T.-T. Chang.
Kalimantan - T. Jung, M. Horta Jung and M. Tarigan.
Sumatra - T. Jung, I. Milenkovic, T. Corcobado, L. Oliveira and M. Tarigan.
Japan - T. Jung, M. Horta Jung, C.M. Brasier, J.F. Webber, K. Kageyama, A. Hieno, H. Masuya and S. Uematsu.

Table S2. Primers used for the confirmation of each virus presence in the collection of '*Phytophthora palustris*' isolates by RT-PCR

Acronym	Name	Sequence	Tm
**PpaBbLV1-1**	BOTY1_SU_FW	GTGGTACGAAGAATACTCCGCTTA	60.2
	BOTY1_SU_RE	TACGCCAAATTGAGTACTGTGTTG	59.8
**PpaBbVL1-2**	BOTY2_SU_FW	GAAAATAAGGACTTAAGACGCCGG	60.0
	BOTY2_SU_RE	CCTCAGTGATACGCATATTCCCTT	60.3
**PpaBbVL1-3**	BOTY3_SU_FW	CTCTTAAATACGATGCGCTACCAC	59.9
	BOTY3_SU_RE	GGAGCCTCTTCTGTTGAAACTAGA	60.0
**PpaBbLV2**	BOTY_KA_RE	CGAACGTGTATCTGACCAAAAGAG	59.9
	BOTY_KA_FW	TTTTATGGCTACAAAAGAAGGGGC	59.8
**PpaBbLV2**	BOTY2_RACE	ATCGCAGTCTTCTCCACCAGGTCGG	68.0
**PpaBLV1**	SU_BUNYA1_FW	CGTTCCACCCATAGTAAGCATCT	60.2
	SU_BUNYA1_RE	AGATGGGTTATGGATTGCAGGAG	60.2
**PpaBLV2**	SU_BUNYA2_FW	ACTTAACTCTTTCCGATGGCTGT	60.0
	SU_BUNYA2_RE	ACAAATGTGATGGAAATGGCTGA	59.2
**PpaBLV3**	SU_BUNYA3_FW	ATGAGTGTCATGGGCAATAGGTT	60.1
	SU_BUNYA3_RE	TACAATTTCGACAAGTTGGTGGC	60.0
**PpaBLV4**	SU_BUNYA4_FW	CACAGGGACACAGGTTTTGATTC	60.0
	SU_BUNYA4_RE	GACACAACACCTTTGCTTACCTC	60.0
**PpaBLV5**	SU_BUNYA5_FW	GAAGCATCTCTGGCATCATTTCC	60.0
	SU_BUNYA5_RE	AAGATATCAAATCCGCAAAGCCG	60.0
**PpaBLV6**	SU_BUNYA6_FW	AAGCACTGATCTAAGGCACTCAA	60.0
	SU_BUNYA6_RE	GGCAGGCAGACACATCATAATTC	60.0
**PpaBLV7**	SU_BUNYA7_FW	CGATTAAACTCGCGACAAAATTCC	59.5
	SU_BUNYA7_RE	TACCGAGCGAGATTGAAAGTGAA	60.1
**PpaBLV8**	SU_BUNYA8_FW	TCATTGCCTAGTTTACAGTTTTCT	56.5
	SU_BUNYA8_RE	ATGTCATTCAGATGATAGTGCCA	57.7
**PpaBLV9-1**	SU_BUNYA9_FW	CTTCGATGTAATCCGGGCTTTTC	60.0
	SU_BUNYA9_RE	GAGCGGAAATGAGATGTTACTGC	60.0
**PpaBLV9-2**	SU_BUNYA11_RE	TTCAGTGGCATCTATGGACAGTT	59.7
	SU_BUNYA11_FW	ATGAAGCTCCAAATTAGGGCGAT	60.4
**PpaBLV9-3**	SU_BUNYA12_FW	GTTGACCTTGTTTAGAACACCGG	60.0
	SU_BUNYA12_RE	CTTGTCGAATTCACCGTGTCAAA	60.0
**PpaBLV10**	SU_BUNYA10_FW	TCGAACACGTTAAGGTGAGACAT	60.0
	SU_BUNYA10_RE	CGAGCAGGCTGATTTCATTAAGG	60.0
**PpaBLV11**	SU_BUNYA13_FW	TCATGACGCTAGATCCGTTTTCT	59.9
	SU_BUNYA13_RE	AGTGTGTTGAACTTAGTCAGCAA	58.5
**PpaBLV11**	SU_BUNYA14_FW	AACACCTCTCTGAAGAGCGAAAT	60.0
	SU_BUNYA14_RE	TCTGCCAGCTCTTGTTTTCAATG	60.0

Acronym	Name	Sequence	Tm
**PpaBLV11**	NP_Bunya_FW	GTTGCCTTCCCACGACAAAC	60.0
	NP_BUNYA_RE	ACTTGTCATGAGCACGAGCA	60.0
**PpaBLV11**	Bunya 11_1,033 F	CCTGATAATATACTTCGTGACTGTCCT	60.0
**PpaBLV12**	KA_BUNYA1_FW	CATCAGCCTTTCATGCAGAGATG	60.0
	KA_BUNYA1_RE	CTCACTTGAATGTATTCGACGGC	60.0
**PpaBLV13-1**	KA_BUNYA2_FW	GACGTAAACTTTGGGAACACAGG	60.0
	KA_BUNYA2_RE	AGCGAACAGACTACTAGTGCAAA	60.0
**PpaBLV13-2**	KA_BUNYA3_RE	GAGACCTTTTCGCATGTTCACAA	60.0
	KA_BUNYA3_FW	TCCTCTCTTTTCGTCATCCATGA	59.2
**PpaBLV13-3**	KA_BUNYA4_RE	GCTCACTTTCGCTTCCTAATGTC	59.9
	KA_BUNYA4_FW	TCCCTTCATCATCTACCTGTGAA	58.6
**PpaNLV1**	SU_NARNA1_FW	CTCGGGAAAAGTTTACACGTGAC	60.1
	SU_NARNA1_RE	ACCTTGTATCCAAAGCGATTCCT	60.1
**PpaNLV2**	SU_NARNA2_FW	CTGATGGCTCAATCGAACACATC	60.0
	SU_NARNA2_RE	GTCGTGACTATCTTACGCAGGAT	60.0
**PpaNLV3**	SU_NARNA3_RE	CAGCATAGTTCCACTCTGTCTC	60.1
	SU_NARNA3_FW	GCGTATTCTGCGAGCAACTAGTAG	61.1
**PpaNLV4**	SU_NARNA4_FW	TCCATCTCCACTAATTGTTCGCA	60.1
	SU_NARNA4_RE	TATCAGCGAACGACGAATGGTAT	60.0
**PpaNLV5**	SU_NARNA5_FW	GAGGACCTTGGGCTAAAGATCAA	60.1
	SU_NARNA5_RE	GAGGTTGGTGGATTCTGAGACTT	60.0
**PpaNLV8**	KA_NARNA3_FW	AGCAAAGAGACGTTCCTGAAGAT	60.0
	KA_NARNA3_RE	AGTGCAAGCCAATGTCTTCAATC	60.1
**PpaNLV7**	KA_NARNA1_FW	CCGATGATGGTGGAAAATGGATG	60.0
	KA_NARNA1_RE	GGTTAAACCAGACTGCAAACTCC	60.0
**PpaNLV6**	SU_NARNA10_FW	TGGCGGTGTTATCATATAGGTGG	60.0
	SU_NARNA10_RE	TCGAAAACCAATGATTGCGTTCA	60.0
**PpaTLV1-1**	KA_TOTI1_FW	GAAAAACTTTCCAACGACGGGAT	60.0
	KA_TOTI1_RE	GCTGTTAATGAATGTCGTAGCCC	60.0
**PpaTLV1-2**	KA_TOTI4_FW	AGCTTGAACATGGAAAAAGTCGG	60.0
	KA_TOTI4_RE	GCTGTTAATGAATGTCGTAGCCC	60.0
**PpaTLV2-1**	KA_TOTI2_FW	GCAGAGCGGATAGTTGAACAATC	60.0
**PpaTLV2-3**	KA_TOTI2_RE	GCCGAGTGTTGTTTGATCTTGAA	60.0
**PpaTLV2-2**	KA_TOTI3_FW	TTCAAGATCAAACAACACTCGGC	60.0
	KA_TOTI3_RE	ACGCTAGCAAAAGTCTGGTCTTA	60.0
**PpaTLV3-1**	KA_TOTI5_FW	AGGAAAGAGGGCTAACGAAACAT	60.0
	KA_TOTI5_RE	CGCATCTCATTCGCTTCAATTCT	60.0
**PpaTLV3-2**	KA_TOTI9_FW	AAGCCTTTCTGTACAACATGTGC	60.0
	KA_TOTI9_RE	ACATCGTCCTCGTATACTTCGTG	60.0
**PpaTL3-3**	SU_TOTI4_FW	TGTGGCTGTGGATTAAGCAGTAT	60.1

	SU_TOTI4_RE	AAAGCCAAAGCCTTGTCATTCTC	60.0
**Acronym**	**Name**	**Sequence**	**Tm**
**PpaTLV4**	SU_TOTI5_RE	AGTAGTGCGCGACATCATAATCT	60.0
	SU_TOTI5_FW	CCTATGTCTCATCTGGCATGGAA	59.9
**PpaTLV5-1**	KA_TOTI13_FW	ATAATGATGTGCTCCACGCCTAT	60.0
	KA_TOTI13_RE	TTCGACTGGCTTAAGTATGTGCT	60.1
**PpaTLV5-2**	SU_TOTI1_RE	ACGTTCGAAACTCTTCACTAGCT	60.0
	SU_TOTI1_FW	CTGGTGCCATGAACAAAGTCAAT	60.0
**PpaTLV6**	KA_TOTI6_FW	CTTATGCGTCTCAATCAATGCGT	60.0
	KA_TOTI6_RE	TGTGAACGTTATTTGCCTTCACC	60.0
**PpaTLV7**	KA_TOTI7_FW	TTCGTATCCTCAGCTTTGGTTCA	60.0
	KA_TOTI7_RE	TGCCATCGAAAAACAGTGAAGTC	60.0
**PpaTLV8**	KA_TOTI8_FW	GGGCTTGGAAGATAAGTCAGTCA	60.1
	KA_TOTI8_RE	GGGATTCTGATCGAAAATGGCTG	60.0
**PpaTLV9**	KA_TOTI10_FW	GCAGACATTATCCTCCCATTTGC	60.0
	KA_TOTI10_RE	AACAGCGTTCTCAACTTAGCCTA	60.0
**PpaTLV10**	KA_TOTI11_FW	CTGCGTTCAGGAACTTGTTCAAT	60.0
	KA_TOTI11_RE	CACAAAGGAGAAAATTGACGGCT	60.0
**PpaTLV11**	KA_TOTI12_FW	ACATGCATATCTAGAAGGCTGGG	60.0
	KA_TOTI12_RE	AATGACCACATTCAGTTGCTTCG	60.1
**PpaTLV12-1**	KA_TOTI14_FW	TTGGGGAGCGTTTTGTCTATGTA	60.0
	KA_TOTI14_RE	GGCTCATCAATCTTACGAAACGG	60.0
**PpaTLV12-2**	SU_TOTI3_RE	TAGTTCTGCATTTGGTTGGCTTG	60.0
	SU_TOTI3_FW	AGTACGGATTGACGGTTGTTACA	60.0
**PpaTLV12-3**	SU_TOTI3_RE	TAGTTCTGCATTTGGTTGGCTTG	60.0
	SU_TOTI3_FW	AGTACGGATTGACGGTTGTTACA	60.0
**PpaTbLV1**	SU_Tombus_RE	TCTTCCATGAACAACAAAGCGTC	60.0
	SU_Tombus_FW	GCCAATGGAAAAGCAGTTTAGGT	60.0
**PpaBLV11**	RACE_NC_FW	GAACTGCCCCTTCGTCCACTTTGCC	68.1
	RACE_NC_RE	GGCAAAGTGGACGAAGGGGCAGTTC	68.1
	RACE_RDRP_RE	TGTCGAGCATTTCCTCAGCGGCTTC	67.4
	RACE_1,563 RE (RDRP)	TGGCTTGCCAAGAGGGGACAGAAAGGA	69.6
	RACE_5,283 FW (RDRP)	GCGCTGTGACCACTTCAGGCTCGAACT	70.6
	RACE_FW_Bunya11 (RDRP)	TGCTTGGCTGTTTTCACATCAGACCCC	67.6

Table S3. Trimmed low quality and adaptor data.

MultiQC Trimmed Data				
**Sample Name**	**Trimmed**	**GC**	**Length (bp)**	**nr Seqs**
JP_TW_R1	3.80	50	144	234,370,395
JP_TW_R2	3.80	50	144	234,370,395
JP1_R1	4.30	50	143	288,057,483
JP1_R2	4.40	50	143	288,057,483
JP2_R1	3.10	50	145	287,382,226
JP2_R2	3.30	50	145	287,382,226
SU1_R1	4.20	50	144	207,472,787
SU1_R2	4.20	51	143	207,472,787
SU2_R1	4.30	51	143	236,535,193
SU2_R2	4.30	51	143	236,535,193
SU3_R1	4.00	51	144	224,042,957
SU3_R2	4.00	51	144	224,042,957
SU4_R1	3.30	48	145	302,757,825
SU4_R2	3.50	48	145	302,757,825
KA1_R1	3.90	50	144	221,656,189
KA1_R2	3.90	50	144	221,656,189
KA2_R1	3.30	49	145	299,814,921
KA2_R2	3.40	49	145	299,814,921
KA3_R1	2.90	49	145	281,646,551
KA3_R2	3.10	49	145	281,646,551

Table S4. Mapped reads to *Phytophthora parasitica* genome (used as a reference host genome)

Sample	Total reads	Mapped reads	%
JP_TW	470,718,504	445,459,810	94.63
JP1	578,962,481	559,162,219	96.58
JP2	578,300,676	570,165,513	98.59
SU1	418,527,380	406,250,977	97.07
SU2	476,484,813	468,200,656	98.26
SU3	450,396,585	414,409,495	92.00
SU4	608,477,112	598,170,418	98.31
KA1	445,391,062	375,964,538	84.41
KA2	601,884,640	580,362,703	96.42
KA3	567,968,429	519,878,953	91.53

Table S5. Data of the resulting contigs obtained by the *de novo* assembling in each sequenced RNA library

Assembly	JP-TW	JP1	JP2	SU1	SU2	SU3	SU4	KA1	KA2	KA3
# **contigs (>= 0 bp)**	54,135	17,128	13,929	49,994	30,077	38,344	24,139	33,127	25,398	29,755
# **contigs (>= 500 bp)**	23,901	34,873	28,483	21,391	13,670	17,625	47,458	11,927	54,143	69,139
# **contigs (>= 1000 bp)**	11,348	8,037	6,909	9,050	6,140	7,970	11,957	4,455	11,109	12,397
# **contigs (>= 5000 bp)**	291	319	312	139	158	178	473	85	391	289
# **contigs (>= 10000 bp)**	28	38	33	4	15	18	72	5	72	35
# **contigs (>= 25000 bp)**	2	2	2	0	0	1	2	0	0	0
# **contigs (>= 50000 bp)**	0	0	0	0	0	0	0	0	0	0
**N50**	1,647	1,632	1,727	1,333	1,482	1,430	1,687	1,228	1,464	1,362
**N75**	938	942	999	831	892	881	984	770	879	835
**L50**	5,755	3,956	3,141	5,683	3,382	4,481	5,619	3,116	6,099	7,550
**L75**	12,203	8,673	6,912	11,642	7,164	9,374	12,184	6,542	13,240	15,928
**GC (%)**	52.95	56.57	54.99	51.82	55.33	54.3	54.51	54.34	54.89	56.3
**Mismatches**										
# **N's**	6,280	34,949	35,617	8,973	9,120	9,940	54,015	4,730	37,512	41,378
# **N's per 100 kbp**	19.81	151.05	179.99	35.96	52.82	45.5	160.51	35.78	116.78	116.76

Pairwise identities of the RdRp nucleotide (**Table S6**) and amino acid (**Table S7**) sequences of '*Phytophthora palustris*' botybirnaviruses

Table S6

	PpaBbV2	PpaBbV1-1	PpaBbV1-2	PpaBbV1-3
PpaBbV2				
PpaBbV1-1	71.62			
PpaBbV1-2	72.44	83.80		
PpaBbV1-3	72.23	82.14	86.75	

Table S7

	PpaBbV2	PpaBbV1-1	PpaBbV1-2	PpaBbV1-3
PpaBbV2				
PpaBbV1-1	81.14			
PpaBbV1-2	81.19	92.36		
PpaBbV1-3	81.92	92.56	94.75	

Table S8. Pairwise identities of the RdRp amino acid sequences of '*Phytophthora palustris*' bunyaviruses

	PpaBLV6	PpaBLV11	PpaBLV5	PpaBLV1	PpaBLV 13-1	PpaBLV 13-2	PpaBLV 13-3	PpaBLV8	PpaBLV2	PpaBLV7	PpaBLV 9-1	PpaBLV 9-3	PpaBLV 9-2	PpaBLV3	PpaBLV4	PpaBLV10	PpaBLV12
**PpaBLV 6**																	
**PpaBLV 11**	9.59																
**PpaBLV 5**	9.80	10.05															
**PpaBLV 1**	10.24	10.19	15.94														
**PpaBLV 13-1**	10.28	10.37	15.74	81.47													
**PpaBLV 13-2**	10.18	10.30	15.74	81.26	92.03												
**PpaBLV 13-3**	10.34	11.06	15.60	83.71	92.70	99.62											
**PpaBLV 8**	9.36	9.64	15.17	28.29	28.54	28.57	31.71										
**PpaBLV 2**	10.77	12.15	18.33	33.98	34.12	33.70	33.70	59.12									
**PpaBLV 7**	9.80	11.06	16.62	30.56	30.33	30.30	32.00	50.75	87.42								
**PpaBLV 9-1**	9.47	11.13	16.61	29.79	29.96	29.54	32.03	51.03	87.60	86.52							
**PpaBLV 9-3**	10.07	11.43	16.79	30.30	30.46	30.23	32.11	51.63	87.60	88.50	94.27						
**PpaBLV 9-2**	9.76	11.21	16.40	29.68	29.74	29.61	31.96	50.62	87.65	86.49	90.85	94.99					
**PpaBLV 3**	9.72	10.33	15.23	27.49	27.71	27.37	29.30	32.17	37.00	33.48	33.13	34.09	33.43				

**PpaBLV 4**	9.75	10.60	15.10	27.52	27.90	27.84	29.37	32.20	36.72	32.97	32.94	33.86	33.08	89.55			
**PpaBLV 10**	9.85	10.65	14.85	27.05	27.45	27.45	30.99	31.31	39.69	34.97	34.77	35.47	34.79	67.60	70.54		
**PpaBLV 12**	9.45	10.55	16.01	29.38	29.73	29.70	31.88	34.58	40.56	35.49	35.70	36.53	35.52	76.63	77.92	73.57	

Table S9. Pairwise identities of the RdRp nucleotide sequences of '*Phytophthora palustris*' bunyaviruses

	**PpaBLV1**	**PpaBLV 13-1**	**PpaBLV 13-2**	**PpaBLV 13-3**	**PpaBLV2**	**PpaBLV7**	**PpaBLV9-1**	**PpaBLV9-2**	**PpaBLV9-3**	**PpaBLV8**	**PpaBLV8**	**PpaBLV3**	**PpaBLV4**	**PpaBLV10**	**PpaBLV12**	**PpaBLV11**	**PpaBLV5**	**PpaBLV6**
**PpaBLV 1**																		
**PpaBLV 13-1**	72.90																	
**PpaBLV 13-2**	72.21	82.05																
**PpaBLV 13-3**	72.12	81.41	99.30															
**PpaBLV 2**	42.49	42.61	42.43	42.51														
**PpaBLV 7**	42.38	42.36	42.83	42.80	73.00													
**PpaBLV 9-1**	42.81	42.40	42.95	42.80	74.29	76.53												
**PpaBLV 9-2**	42.43	42.02	42.36	42.23	73.74	75.88	79.13											
**PpaBLV 9-3**	42.95	42.53	42.80	42.64	73.53	76.73	84.14	90.68										
**PpaBLV 8**	41.00	40.58	41.16	41.04	53.97	53.91	53.87	53.71	54.20									
**PpaBLV 8**	40.96	40.53	41.12	41.00	53.90	53.84	53.81	53.65	54.13	99.88								
**PpaBLV 3**	40.91	40.91	40.93	40.79	45.69	45.33	46.18	46.11	46.00	45.24	45.18							

**PpaBLV 4**	40.89	41.01	40.99	40.85	45.78	45.51	46.23	45.81	46.22	45.16	45.10	85.31					
**PpaBLV 10**	41.15	41.18	41.70	41.58	45.35	45.12	45.73	45.22	45.85	45.44	45.38	74.34	78.47				
**PpaBLV 12**	41.12	41.18	41.53	41.63	45.51	45.54	45.84	45.54	45.62	44.89	44.84	74.51	74.98	75.37			
**PpaBLV 11**	25.81	25.49	25.47	25.40	27.63	27.12	27.31	26.78	27.04	25.21	25.18	26.23	26.24	25.98	26.16		
**PpaBLV 5**	31.67	31.66	31.75	31.74	33.07	33.01	33.08	32.94	33.16	31.40	31.37	31.83	31.82	31.63	31.51	23.07	
**PpaBLV 6**	23.61	23.63	23.86	23.92	24.73	24.62	24.76	24.42	24.91	23.25	23.25	24.47	24.33	24.30	24.36	21.13	21.91

Table S10. Pairwise identities of the RdRp nucleotide sequences of '*Phytophthora palustris*' putative narnaviruses

	**PpaNLV1**	**PpaNLV7**	**PpaNLV2**	**PpaNLV5**	**PpaNLV6**	**PpaNLV4**	**PpaNLV8**	**PpaNLV3- 1**	**PpaNLV3-2**	**PpaNLV3- 3**
**PpaNLV1**										
**PpaNLV7**	27.14									
**PpaNLV2**	27.42	26.30								
**PpaNV5**	27.67	25.93	35.14							
**PpaNLV6**	19.07	16.84	21.04	20.90						
**PpaNLV4**	29.49	27.90	35.01	33.98	20.30					
**PpaNLV8**	14.81	11.91	16.78	16.67	18.11	22.30				
**PpaNLV3- 1**	31.68	29.11	35.64	34.62	21.08	81.48	24.17			
**PpaNLV3- 2**	31.41	29.14	35.84	34.52	21.08	80.96	25.33	93.16		
**PpaNLV3- 3**	31.36	29.17	35.84	34.42	20.83	80.68	25.64	92.92	99.08	

*PpaNLV 6 and 7 have surely partial sequences

Table S11. Pairwise identities of the RdRp amino acid sequences of '*Phytophthora palustris*' narnaviruses

	PpaNLV6	PpaNLV8	PpaNLV4	PpaNLV3- 1	PpaNLV3-2	PpaNVL3-3	PpaNLV2	PpaNLV5	PpaNLV1	PpaNLV7
**PpaNLV6**										
**PpaNLV8**	5.44									
**PpaNLV4**	8.89	33.33								
**PpaNLV3-1**	8.88	34.48	85.95							
**PpaNLV3-2**	8.42	35.09	85.44	95.53						
**PpaNLV3-3**	8.57	35.09	84.41	94.54	99.31					
**PpaNLV2**	8.92	15.97	22.08	21.99	22.61	21.86				
**PpaNLV5**	7.21	25.38	24.38	24.38	24.26	24.38	39.02			
**PpaNLV1**	8.20	9.21	12.10	12.50	12.61	12.49	11.81	7.26		
**PpaNV7**	8.62	6.93	11.61	11.53	11.61	11.52	10.58	7.52	15.15	

Table S12. Pairwise identities of the RdRp nucleotide sequences of '*Phytophthora palustris*' toti-like viruses

	PpaTL V 3-3	PpaTL V 3-1	PpaTL V 3-2	PpaTL V 13-1	PpaTL V 13-2	PpaTL V 13-3	PpaTL V 11	PpaTL V5-1	PpaTL V5-2	PpaTL V10	PpaT LV6	PpaT LV 8	PpaT LV 4	PpaT LV7	PpaTL V2-1	PpaTL V2-2	PpaTL V 2- 3	PpaTL V 12	PpaTL V 1-1	PpaTL V1-2
**PpaTL V 3-3**																				
**PpaTL V 3-1**	83.12																			
**PpaTL V 3-2**	84.28	83.79																		
**PpaTL V 12-1**	25.03	25.04	25.06																	
**PpaTL V 12-2**	22.98	22.88	22.97	75.73																
**PpaTL V 12-3**	22.85	22.68	22.77	76.08	94.26															
**PpaTL V 10**	25.33	25.12	24.87	25.14	24.91	24.75														
**PpaTL V5-1**	26.70	26.50	26.43	26.79	25.63	25.96	33.02													

**PpaTL V5-2**	26.72	26.35	26.17	26.51	25.37	25.85	32.60	82.67												
**PpaTL V9**	26.59	26.72	26.68	25.31	25.28	24.93	31.46	34.00	33.72											
**PpaTL V6**	25.37	25.13	25.38	25.88	25.64	25.64	27.50	28.91	28.61	30.38										
**PpaTL V 8**	22.25	21.82	22.26	21.76	21.74	21.79	22.79	24.75	24.71	24.23	25.91									
**PpaTL V 4**	13.69	13.67	13.76	12.19	11.57	11.57	11.45	12.50	12.16	11.57	11.32	13.40								
**PpaTL V7**	21.88	21.60	21.58	22.01	23.92	24.29	22.38	24.61	25.03	23.54	24.39	41.33	11.69							
**PpaTL V2-1**	29.14	28.37	28.53	27.75	27.81	27.46	34.94	37.93	37.39	35.61	33.40	26.99	13.54	25.83						
**PpaTL V2-2**	29.07	28.65	29.00	28.27	27.88	27.72	35.50	38.73	37.25	36.16	33.40	27.11	14.00	26.06	84.75					
**PpaTL V 2- 3**	29.21	28.71	28.93	28.46	28.03	27.94	35.36	38.69	37.27	36.09	33.45	27.26	14.04	26.14	84.99	96.35				
**PpaTL V 11**	26.20	25.23	25.77	26.19	26.16	26.21	32.87	32.65	32.76	31.88	30.32	23.50	12.06	24.14	38.28	38.38	38.48			
**PpaTL V 1-1**	27.56	26.31	26.18	26.75	29.17	29.05	34.88	34.80	33.93	32.91	30.93	25.00	13.59	27.10	43.62	43.68	43.92	38.11		
**PpaTL V1-2**	27.29	26.06	25.93	26.72	29.01	28.80	34.56	34.56	33.66	32.63	30.64	24.80	13.59	26.92	43.19	43.24	43.46	37.81	97.75	

Table S13. Pairwise identities of the RdRp amino acid sequences of '*Phytophthora palustris*' toti-like viruses.

	PpaTLV 12-1	PpaTLV 12-2	PpaTLV 12-3	PpaTL V4	PpaTL V7	PpaTL V8	PpaTL V3-1	PpaTL V3-2	PpaTL V3-3	PpaTL V10	PpaTL V6	PpaTV L11	PpaTL V1	PpaTL V2-1	PpaTL V2-2	PpaTL V2-3	PpaTL V9	PpaTL V5-1	PpaTL V5-2
**PpaTLV 12-1**																			
**PpaTLV 12-2**	95.58																		
**PpaTLV 12-3**	95.04	98.39																	
**PpaTLV 4**	11.44	11.09	10.92																
**PpaTLV 7**	10.38	10.38	10.26	86.06															
**PpaTLV 8**	11.64	11.49	11.34	86.65	96.73														
**PpaTLV 3-1**	10.41	10.30	10.52	12.52	10.20	12.36													
**PpaTLV 3-2**	10.85	10.74	10.85	13.04	10.88	13.29	92.20												
**PpaTLV 3-3**	10.29	10.18	10.40	13.04	10.42	12.82	93.34	94.08											
**PpaTLV 10**	10.53	10.85	10.64	12.84	11.58	13.88	14.61	14.81	15.14										
**PpaTLV 6**	11.10	10.88	10.78	14.78	12.29	15.79	14.31	14.08	14.19	22.25									
**PpaTVL 11**	11.10	10.79	10.79	14.76	11.78	15.37	13.72	14.03	13.92	28.00	24.58								
**PpaTLV 1**	12.28	12.06	11.84	13.84	12.02	15.06	15.82	16.15	15.92	30.00	27.86	35.11							
**PpaTLV 2-1**	12.75	12.42	12.42	15.68	13.63	16.50	15.25	15.59	15.47	29.21	28.40	32.65	48.84						
**PpaTLV 2-2**	12.69	12.32	12.32	15.76	13.80	16.46	15.77	16.26	16.02	28.46	29.19	33.11	50.07	95.32					
**PpaTLV 2-3**	12.68	12.32	12.32	15.76	13.80	16.46	15.65	16.13	15.89	28.45	29.31	33.10	50.06	95.44	99.49				
**PpaTLV 9**	10.79	11.20	11.20	14.18	11.47	14.72	14.78	15.20	15.10	27.11	25.40	27.97	31.30	31.22	31.95	31.94			

**PpaTLV 5-1**	11.97	11.86	11.86	14.10	12.17	14.67	15.23	16.13	15.68	27.29	26.63	27.90	32.87	36.50	36.74	36.73	32.58		
**PpaTLV 5-2**	12.56	12.56	12.56	13.94	12.06	14.38	15.90	16.97	16.61	26.27	27.10	28.79	34.29	37.19	37.47	37.47	33.52	89.19	

Table S14. Pairwise identities of the CP amino acid sequences of '*Phytophthora palustris*' toti-like viruses.

	**PpaTLV8**	**PpaTLV3-1**	**PpaTLV3-2**	**PpaTLV3-3**	**PpaTLV 12-1**	**PpaTLV 12-2**	**PpaTLV 12-3**	**PpaTLV7**	**PpaTLV6**	**PpaTLV5-1**	**PpaTLV5-2**	**PpaTLV9**	**PpaTLV10**	**PpaTLV11**	**PpaTLV1-1**	**PpaTLV1-2**	**PpaTLV2-1**	**PpaTLV2-2**	**PpaTLV2-3**
**PpaTLV 8**																			
**PpaTLV 3-1**	6.89																		
**PpaTLV 3-2**	6.89	97.22																	
**PpaTLV 3-3**	6.89	97.95	97.95																
**PpaTLV 12-1**	8.21	7.20	7.58	7.33															
**PpaTLV 12-2**	7.88	7.35	7.72	7.48	87.55														
**PpaTLV 12-3**	7.98	7.11	7.48	7.23	87.81	97.37													
**PpaTLV 7**	7.84	7.26	6.93	7.04	7.38	7.84	7.82												
**PpaTLV 6**	10.08	9.07	9.19	8.95	9.38	9.54	9.51	10.53											
**PpaTLV 5-1**	9.51	9.43	9.69	9.43	9.16	9.31	9.40	9.13	18.57										
**PpaTLV 5-2**	9.39	9.69	9.94	9.69	9.78	9.93	9.89	8.91	18.70	95.05									
**PpaTLV 9**	8.53	10.55	10.55	10.67	10.16	10.46	10.66	10.67	17.40	22.56	23.08								
**PpaTLV 10**	10.32	10.51	10.76	10.51	8.54	9.34	9.55	10.09	17.80	22.05	22.31	22.14							

**PpaTLV 11**	8.88	10.17	10.42	10.29	11.30	11.22	11.30	10.88	20.63	23.49	24.01	26.51	30.36						
**PpaTLV 1-1**	10.01	11.65	11.90	11.65	11.23	11.64	11.60	12.40	19.43	24.49	24.75	25.77	29.45	32.09					
**PpaTLV 1-2**	10.01	11.65	11.90	11.65	11.23	11.64	11.60	12.40	19.43	24.49	24.75	25.77	29.45	32.09	100.00				
**PpaTLV 2-1**	8.99	10.63	10.50	10.50	11.84	12.48	12.56	11.80	18.27	25.48	25.48	24.11	29.13	30.99	39.36	39.36			
**PpaTLV 2-2**	9.23	10.88	10.75	10.75	11.59	12.24	12.32	11.69	18.27	24.97	24.97	23.98	29.39	30.87	39.74	39.74	96.55		
**PpaTLV 2-3**	9.23	11.00	10.88	10.88	11.59	12.24	12.32	11.69	18.27	25.22	25.22	23.98	29.39	30.87	39.87	39.87	96.29	99.36	

Table S15. Average pairwise (PW) identity of the alignment of the partial viral sequences (variants) obtained by RT-PCR with the virus contig sequences obtained by NGS

'Phytopthora palustris' virus	PW Identity (%)	Hosts of the virus variants
Botybirna-like 1	87.6	SU1474, SU0158, SU0376
Botybirna-like 2	99.7	KA0139, KA0146, KA0156, KA0119
Bunya-like 1	100.0	SU0043
Bunya-like 2	99.7	SU0043, SU0315, SU0317
Bunya-like 3	99.9	SU0696, SU0431, SU0376, SU0575
Bunya-like 4	88.4	SU0401, SU0361bTRUE, SU0376
Bunya-like 5	100.0	SU0399
Bunya-like 6	100.0	SU0409
Bunya-like 7	99.9	SU0264, SU0401
Bunya-like 8	98.8	SU0399
Bunya-like 9	87.7	SU0344, SU0376, SU0656, SU0141
Bunya-like 10	99.3	SU0095, SU0696, SU0618
Bunya-like 11_NC	100.0	SU1474
Bunya-like 11_RdRp	99.5	SU1474, SU0250, SU0344, SU0376, SU0656
Bunya-like 12	99.7	KA0106
Bunya-like 13	90.8	KA0187, KA0106, KA0142
Narna-like 1	100	SU0046
Narna-like 2	99.4	SU0095, SU0135, SU0158, SU0344, SU0401, SU0531, SU0696
Narna-like 3	94.3	KA0106, KA0120, SU0135, SU0158, SU0401
Narna-like 4	99.9	SU0325, SU0317, SU0318
Narna-like 5	100.0	SU0696
Narna-like 6	99.9	SU1474
Narna-like 7	99.9	KA0119, KA0156, KA0139, KA0146
Narna-like 8	95.6	KA0120
Tombus-like 1	99.8	SU0043, SU0317
Toti-like 1	99.7	KA0119, KA0156, KA0139, KA0146
Toti-like 2	90.7	KA0156, KA0139, KA0147
Toti-like 3	90.2	KA0092, KA0119, KA0156, KA0139, KA0146
Toti-like 4	100.0	SU0376
Toti-like 5	90.9	KA0119, KA0139, KA0146, SU0777, SU0875, SU0987, SU1474, SU1477
Toti-like 6	99.9	KA0119, KA0156, KA0139, KA0146
Toti-like 7	99.8	KA0119, KA0156, KA0139, KA0146
Toti-like 8	99.4	KA0119, KA0156, KA0139, KA0146
Toti-like 9	99.9	KA0119, KA0156, KA0139, KA0146
Toti-like 10	99.9	KA0119, KA0139, KA0146
Toti-like 11	99.9	KA0046, KA0052, KA0059, KA0081, KA0092, KA0102, KA0116, KA0119, KA0120, KA0139, KA0146, KA0156
Toti-like 12	91.7	KA0146, KA0156, SU0015, SU0235, SU0982, SU1474
